# Map3k8 controls granulocyte colony-stimulating factor production and neutrophil precursor proliferation in lipopolysaccharide-induced emergency granulopoiesis

**DOI:** 10.1038/s41598-017-04538-3

**Published:** 2017-07-10

**Authors:** Ángela Sánchez, Carlos Relaño, Araceli Carrasco, Constanza Contreras-Jurado, Antonio Martín-Duce, Ana Aranda, Susana Alemany

**Affiliations:** 1 Instituto de Investigaciones Biomédicas “Alberto Sols” Madrid, Consejo Superior de Investigaciones Científicas (CSIC-UAM), Madrid, Spain; 2 Centro de Investigación Biomédica en Red de Cáncer (CIBERONC), Madrid, Spain; 3 Unidad Asociada de Biomedicina (UA, CSIC), University Palmas de Gran Canaria, Las Palmas, Spain

## Abstract

Map3k8 has been proposed as a useful target for the treatment of inflammatory diseases. We show here that during lipopolysaccharide-induced emergency granulopoiesis, Map3k8 deficiency strongly impairs the increase in circulating mature (Ly6G^high^CD11b^+^) and immature (Ly6G^low^CD11b^+^) neutrophils. After chimaeric bone marrow (BM) transplantation into recipient Map3k8^−/−^ mice, lipopolysaccharide treatment did not increase circulating Ly6G^high^CD11b^+^ cells and strongly decreased circulating Ly6G^low^CD11b^+^ cells. Lipopolysaccharide-treated Map3k8^−/−^ mice showed decreased production of granulocyte colony-stimulating factor (G-CSF), a key factor in neutrophil expansion, and a Map3k8 inhibitor blocked lipopolysaccharide-mediated G-CSF expression in endothelial cell lines. Ly6G^low^CD11b^+^ BM cells from lipopolysaccharide-treated Map3k8^−/−^ mice displayed impaired expression of CCAAT-enhancer-binding protein β, which depends on G-CSF for expression and is crucial for cell cycle acceleration in this life-threatening condition. Accordingly, lipopolysaccharide-treated Map3k8^−/−^ mice showed decreased Ly6G^low^CD11b^+^ BM cell proliferation, as evidenced by a decrease in the percentage of the most immature precursors, which have the highest proliferation capacity among this cell population. Thus, Map3k8 expression by non-haematopoietic tissue is required for lipopolysaccharide-induced emergency granulopoiesis. The novel observation that inhibition of Map3k8 activity decreases neutrophilia during life-threatening systemic infection suggests a possible risk in the proposed use of Map3k8 blockade as an anti-inflammatory therapy.

## Introduction

Haematopoiesis is a tightly regulated, hierarchically organized process for maintaining appropriate numbers of immune cells. Haematopoiesis occurs in the bone marrow (BM) in stages, with each successive stage further restricting lineage choices and decreasing self-renewal capacity^1, 2^. Myeloid haematopoiesis begins with haematopoietic stem cells and progresses through multipotent progenitors and common myeloid progenitors to oligopotent granulocyte-monocyte progenitors (GMPs), which differentiate into monocyte and granulocyte lineage-committed progenies. Neutrophil maturation begins with the stepwise differentiation of GMPs and generates granulocyte precursors that gradually acquire lineage-specific features^3–5^. Emergency granulopoiesis is triggered in response to life-threatening systemic infection. Pathogen-associated molecular patterns instruct the haematopoietic system to generate and mobilize an increasing number of myeloid cells, mostly granulocytes, at the expense of generating other differentiated cell lineages^5, 6^. Pathogen dissemination is sensed by the interaction of pathogen-associated molecular patterns with their cognate receptors, including Toll-like receptors (TLRs), such as TLR4; these receptors play a key role in initiating the response to infection with gram-negative bacteria, which have cell walls containing large amounts of lipopolysaccharide (LPS)^7^. TLR4 has been characterized in the most detail in mature myeloid effector cells, but this receptor is also found in haematopoietic stem and progenitor cells (HSPCs) and in non-haematopoietic cells. TLR4 activation by LPS directly triggers emergency myelopoiesis, primarily through the production of promyeloid signals predominantly involving cytokines, which instruct cells of the myeloid lineage, including HSPCs and immature granulocytes, to proliferate and differentiate into mature myeloid cells^5, 6, 8, 9^. Granulocyte colony-stimulating factor (G-CSF), the principal cytokine controlling homeostatic neutrophil development and function^10^, also affects emergency granulopoiesis^5, 6, 8, 9, 11^. G-CSF increases the production of CCAAT-enhancer-binding protein β (C/EBPβ the major transcriptional regulator of emergency granulopoiesis, which controls the amplification and differentiation of GMPs and immature neutrophils^4–6, 12, 13^. Other cytokines, such as interleukin (IL)-1, tumour necrosis factor (TNF)-α, IL-6, and interferons, are also involved in emergency granulopoiesis, primarily through induction of HSPC proliferation and differentiation^3, 6, 14–21^.

Map3k8 (mitogen-activated protein kinase kinase kinase 8), also known as Cot/tpl2, is crucial for both innate and adaptive immune responses. Map3k8 signalling has been studied primarily downstream of TLR4 signalling in macrophages, where it activates the Map2k1/2-Mapk1/2 pathway and participates in modulating other signal transduction pathways, such as those mediated by c-jun kinase and p70 S6 kinase^22–25^. Map3k8 is also involved in intracellular signalling by TNF-α, IL-1, adiponectin, IL-17, antigen receptors and G protein-coupled receptors^26–31^, thereby regulating the production of inflammatory, M1, and M2 cytokines, such as TNF-α, IL-1β, IL-6, IL-12, IL-10, and interferons, in haematopoietic cells^22–24, 28, 32–34^. Moreover, Map3k8 is essential for mounting an effective immune response during infection. Map3k8^−/−^ mice are more susceptible to *Toxoplasma gondii* ^33^ *, Listeria monocytogenes* ^32^, Group B *Streptococcus* ^35^ *, Mycobacterium tuberculosis* ^36^, influenza virus^31^ and *Schistosoma mansoni* ^19^ infections than wild-type (Wt) mice. Owing to its role in controlling cytokine and chemokine production during inflammatory processes, Map3k8 has been identified as a potentially interesting target for the treatment of various diseases, including autoimmune and liver diseases and diverse types of cancer^37–41^.

We show here that Map3k8 activity controls the massive production of neutrophils in LPS-induced emergency granulopoiesis. Map3k8 regulates LPS-induced G-CSF production, which upregulates C/EBPβ expression in immature neutrophils to trigger their amplification.

## Results

### Map3k8 activity controls LPS-induced emergency granulopoiesis

**Figure 1:**
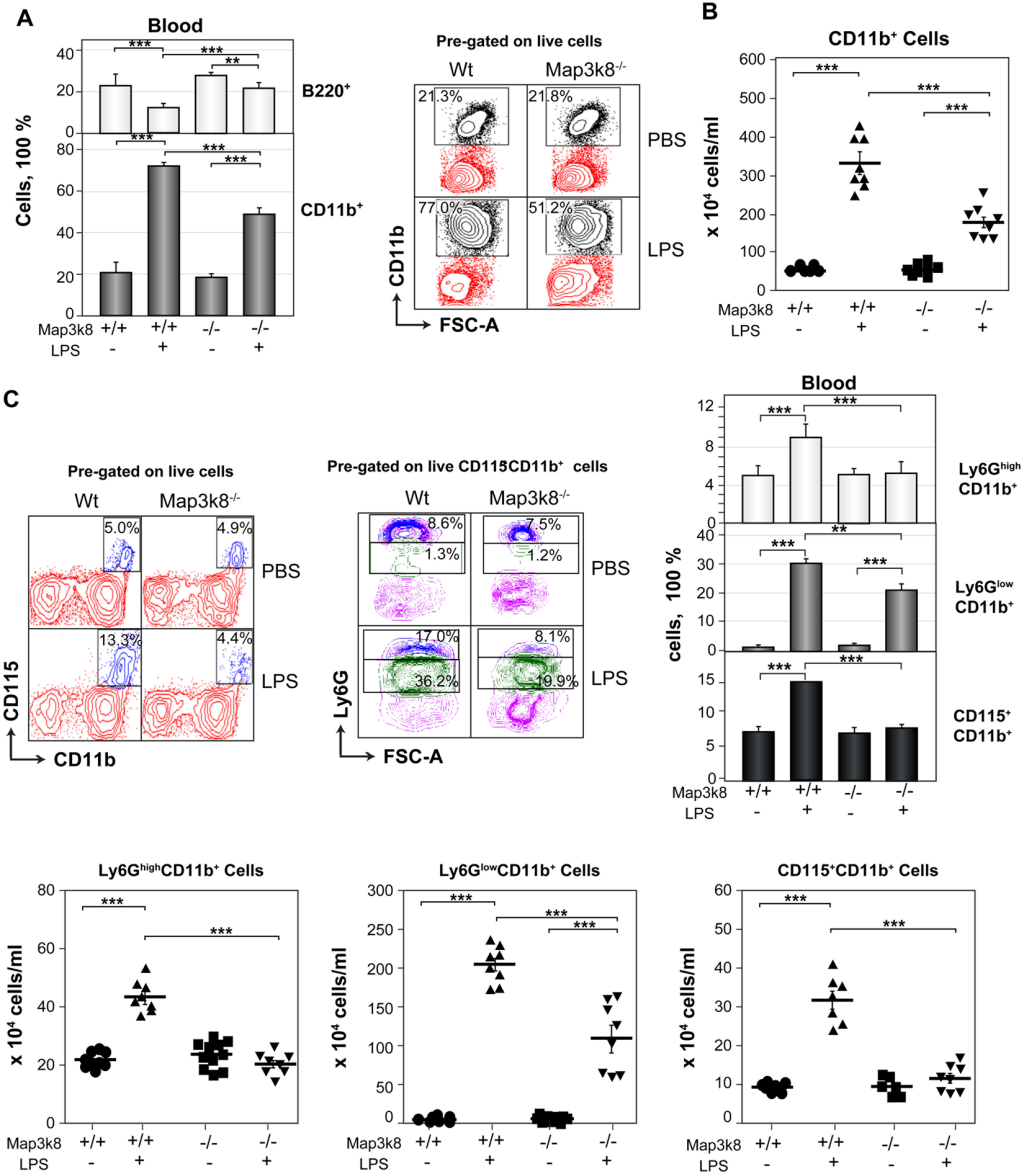
Map3k8 deficiency decreases circulating myeloid cells in LPS-induced emergency haematopoiesis. Wt and Map3k8^−/−^ mice received two i.p. injections of LPS or PBS; 24 h after the last injection, the blood levels of B220^+^ B and CD11b^+^ myeloid cells, Ly6G^high^CD11b^+^ mature and Ly6G^low^CD11b^+^ immature neutrophils, and CD115^+^CD11b^+^ monocytes were analysed by flow cytometry. (**A**) The left panel shows the frequency of B220^+^ B and CD11b^+^ myeloid cells relative to the total number of circulating cells. The right panel shows a representative dot plot depicting circulating CD11b^+^ myeloid cells. (**B**) Number of circulating CD11b^+^ myeloid cells in the different groups. (**C**) Representative dot plots showing the percentage of circulating CD115^+^CD11b^+^ monocytes and of Ly6G^high^CD11b^+^ mature and Ly6G^low^CD11b^+^ immature neutrophils relative to the total number of circulating cells. The right panel shows the percentages of these cells relative to total circulating cells in blood, and the numbers are provided in the lower panel. (**A**–**C**) Mean ± SEM (*n* = 8). Newman-Keuls tests were performed to analyse significant differences among groups. *p < 0.05, **p < 0.01, ***p < 0.001.

Severe systemic infection was mimicked by the intraperitoneal (i.p.) injection of LPS into Wt and Map3k8^−/−^ mice on two consecutive days. We evaluated circulating CD11b^+^ myeloid cells and B220^+^ B cells after 24 h using flow cytometry (see Supplemental Figure S1). The frequency and number of circulating CD11b^+^ cells increased in LPS-treated Wt and Map3k8^−/−^ mice, but these increases were larger in Wt mice than in Map3k8^−/−^ mice (Fig. 1A,B), resulting in a smaller decrease in the percentage of B220^+^ cells in Map3k8^−/−^ mice (Fig. 1A). In agreement with previous findings^8, 9^, the various circulating CD11b^+^ myeloid cell populations—Ly6G^high^CD11b^+^ mature neutrophils, Ly6G^low^CD11b^+^ immature neutrophils, and CD115^+^CD11b^+^ monocytes—increased in LPS-injected Wt mice. However, in Map3k8^−/−^ mice, the percentage and number of Ly6G^high^CD11b^+^ neutrophils and CD115^+^CD11b^+^ monocytes scarcely increased in response to LPS challenge. In addition, the number and percentage of Ly6G^low^CD11b^+^ neutrophils were smaller in Map3k8^−/−^ mice than in their Wt counterparts (Fig. 1C).

We also analysed the myeloid cell populations in the blood of LPS-treated Map3k8^KD^ mice, which express a kinase-defective Map3k8^41^. Similar to Map3k8^−/−^ mice, LPS-treated Map3k8^KD^ mice had a higher percentage of B220^+^ cells and a smaller increase in circulating CD11b^+^ myeloid cells than LPS-treated Wt mice, primarily due to the decreased proportion of Ly6G^low^CD11b^+^ cells and the lack of increases in Ly6G^high^CD11b^+^ and CD115^+^CD11b^+^ cell numbers (see Supplemental Figure S2). We analysed the possibility that Map3k8 regulates the apoptosis rate of circulating myeloid cells. In both Wt and Map3k8^−/−^ mice, LPS treatment increased Annexin V labelling, but there was a higher frequency of Annexin V-positive cells among Ly6G^low^CD11b^+^ and CD115^+^CD11b^+^ cell populations lacking Map3k8. The frequency of Annexin V plus 7-aminoactinomycin D double-labelling was also higher among Map3k8^−/−^ Ly6G^high^CD11b^+^ mature cells than their Map3k8-positive counterparts (see Supplemental Figure S3).

### Map3k8 deficiency decreases the frequency of immature neutrophils in the BM during LPS-induced emergency granulopoiesis

**Figure 2:**
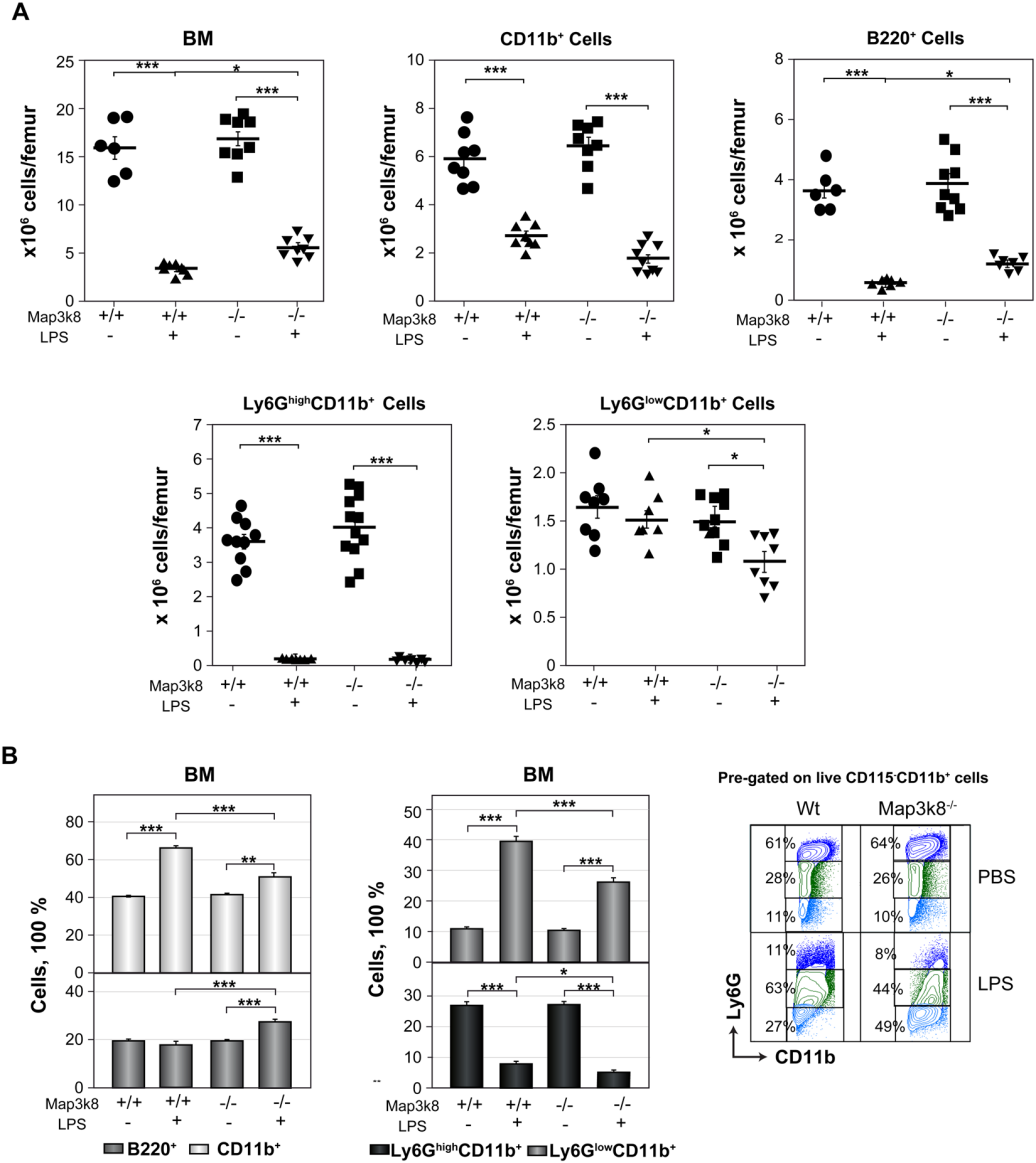
Analysis of BM cells in LPS-treated Wt and Map3k8^−/−^ mice. Wt and Map3k8^−/−^ mice received two injections of LPS or PBS; 24 h after the last injection, BM cells were isolated and subjected to flow cytometry analysis. (**A**) Total cellularity of BM and numbers of BM CD11b^+^ myeloid and B220^+^ B cells and of Ly6G^high^CD11b^+^ mature and Ly6G^low^CD11b^+^ immature neutrophils. (**B**) Frequency of the cells described in (**A**) relative to the total number of BM cells. On the right, representative dot plots show the percentage of Ly6G^high^CD11b^+^ mature and Ly6G^low^CD11b^+^ immature neutrophils relative to the total number of BM CD115^−^CD11b^+^ myeloid cells. The data are presented as the mean ± SEM from at least 3 independent experiments performed in duplicate. Newman-Keuls tests were performed to analyse significant differences among groups. *p < 0.05, **p < 0.01, ***p < 0.001.

BM cellularity decreases in mice after LPS treatment^9^, and we observed that Map3k8 deficiency limited this decrease (Fig. 2A). Compared to their Wt counterparts, LPS-treated Map3k8^−/−^ mice had a similar number of BM CD11b^+^ cells but a smaller decrease in the number of BM B220^+^ cells (Fig. 2A). An analysis of Ly6G-positive cells revealed that Map3k8 deficiency decreased the absolute number of BM Ly6G^low^CD11b^+^ immature cells but not of Ly6G^high^CD11b^+^ mature cells upon LPS treatment. In addition, the frequency of BM CD11b^+^, Ly6G^high^CD11b^+^, and Ly6G^low^CD11b^+^ cells was decreased in LPS-injected Map3k8-deficient mice compared to their Wt counterparts (Fig. 2B). GMPs are direct precursors of cells committed to the monocyte and neutrophil lineages. The increased production of interferon γ, TNF-α, and IL-6 during disseminated infections, such as systemic *E. coli* infection, and after treatment with LPS shifts GMPs (Lineage(LIN)^−^CD117^+^Sca-1^−^CD16/32^+^CD34^+^) to a cell population in which Sca-1 expression is maintained (LIN^−^CD117^+^Sca-1^+^CD16/32^+^CD34^+^) through inversion of the Sca-1^−^ phenotype and enhanced mitosis of Sca-1^+^ cells^2, 5, 18, 42, 43^. To evaluate GMPs, LIN^−^ cells were purified from the BM and subjected to flow cytometry (see Supplemental Figure S1). After LPS treatment, the number of Sca-1^−^ GMPs decreased similarly in the BM of Wt and Map3k8^−/−^ mice, but the increase in the number of Sca-1^+^ GMPs was limited by Map3k8 deficiency (see Supplemental Figure S4).

### Map3k8 disruption decreases the progression of immature Ly6G^low^CD11b^+^ cells through the cell cycle in LPS-treated mice

**Figure 3:**
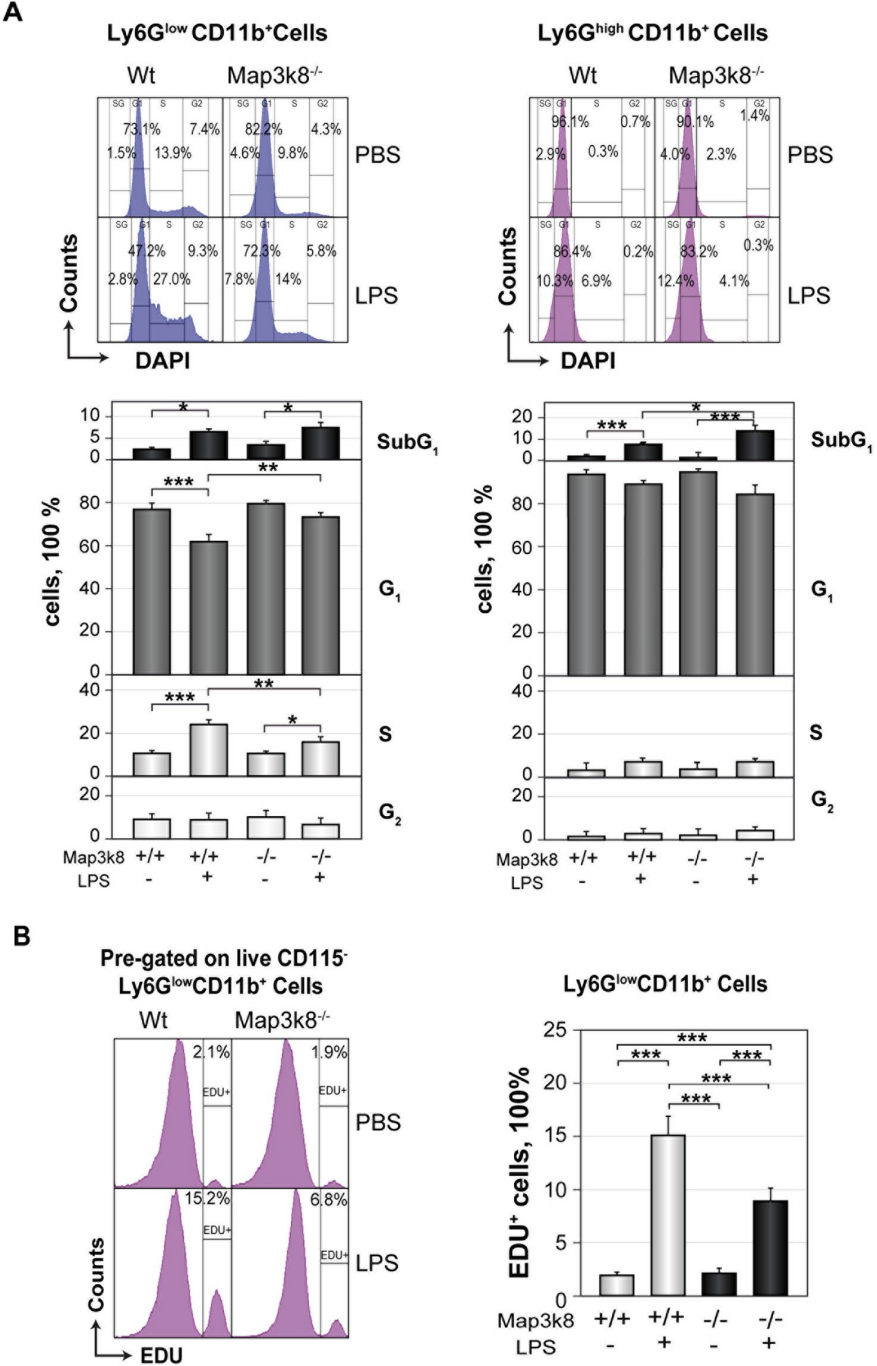
Map3k8 deficiency slows the cell cycle progression of BM Ly6G^low^CD11b^+^ immature neutrophils in LPS-treated mice. (**A**) BM cells from Wt and Map3k8^−/−^ untreated and LPS-treated mice were surface-stained, permeabilized and stained with DAPI. Representative histograms depicting the cell cycle distribution of Ly6G^low^CD11b^+^ immature and Ly6G^high^CD11b^+^ mature neutrophils. The lower panels show the percentage of cells in the different phases of the cell cycle. Data from at least 3 independent experiments performed in duplicate are shown. (**B**) Another set of mice treated as in (**A**) received an injection of 2′-deoxy-5-ethynyluridine (EDU) 18 h before they were killed. BM cells were surface-stained, permeabilized and subjected to the Click-iT® reaction. Histograms depict a representative analysis of EDU-positive cells among the Ly6G^low^CD11b^+^ immature neutrophil population. The graph on the right shows the frequency of EDU-positive cells among Ly6G^low^CD11b^+^ immature neutrophils. Data from two independent experiments performed in triplicate are presented as the mean ± SEM. Newman-Keuls tests were used to compare groups. *p < 0.05, **p < 0.01, ***p < 0.001.

An analysis of the cell cycle in Ly6G^low^CD11b^+^ BM neutrophils showed that LPS treatment increased the frequency of cells in S phase in Wt mice, but this increase was blunted in Map3k8^−/−^ mice (Fig. 3A). LPS treatment resulted in a strongly induced 2′-deoxy-5-ethynyluridine incorporation in Ly6G^low^CD11b^+^ BM cells from Wt mice, but this response was markedly decreased in Map3k8^−/−^ mice (Fig. 3B). Most of the mature Ly6G^high^CD11b^+^ cells from untreated Wt and Map3k8^−/−^ mice were in G_1_ phase. After LPS treatment, we observed an increase in the frequency of SubG_1_-phase mature and immature neutrophils, and this response was partly dependent on Map3k8 expression in Ly6G^high^CD11b^+^ cells (Fig. 3A). These data indicated that Map3k8 contributes to the progression of neutrophil precursors through the cell cycle during LPS-induced granulopoiesis.

### Map3k8 deficiency decreases the percentage of BM myelocytes and metamyelocytes within the Ly6G^low^CD11b^+^ cell population in LPS-treated mice

**Figure 4:**
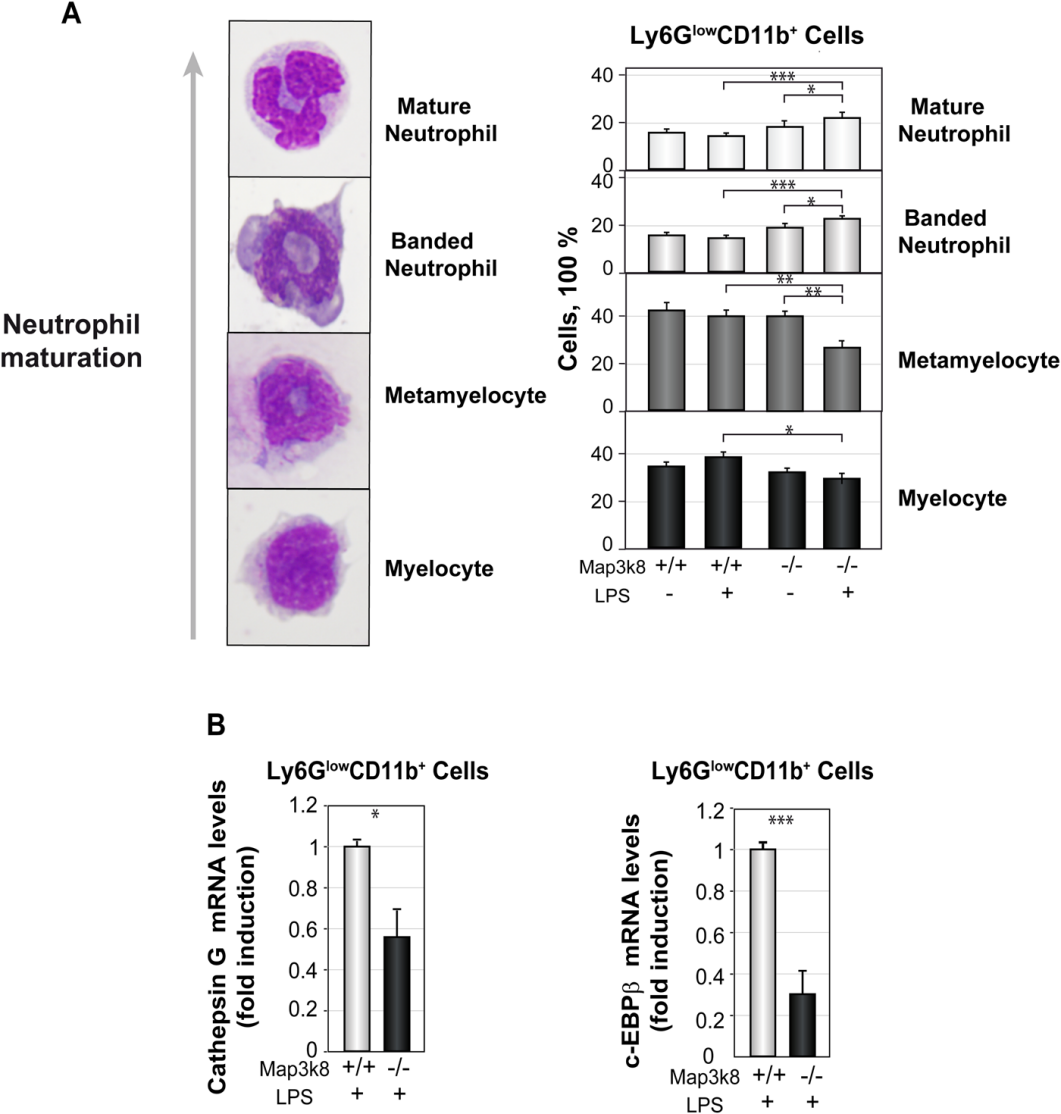
Analysis of BM Ly6G^low^CD11b^+^ immature neutrophils from LPS-treated Wt and Map3k8^−/−^ mice. (**A**) Representative images of cells at different stages of neutrophil maturation are shown at the left. Sorted BM Ly6G^low^CD11b^+^ immature neutrophils from non-treated and LPS-treated Wt and Map3k8^−/−^ mice were subjected to cytospin centrifugation and Wright-Giemsa staining. The percentages of the various neutrophil precursors in the cell populations were scored by two different researchers who were blinded to the groups. The proportions of myelocytes, metamyelocytes, and banded and mature neutrophils within the sorted BM Ly6G^low^CD11b^+^ neutrophil population are shown. Data are presented as the mean ± SEM from three different experiments performed in triplicate. Newman-Keuls tests were used to compare groups. (**B**) Cathepsin G and C/EBPβ mRNA levels in the sorted Ly6G^low^CD11b^+^ immature neutrophils from BM of LPS-treated mice were determined by RT-qPCR. Expression was analysed after normalization to S18. The graphs show the mean ± SEM of three different experiments, each conducted on a pool of cells from three animals. Two-tailed Student’s *t*-tests were used for comparisons of two groups. (**A**,**B**) *p < 0.05, **p < 0.01, ***p < 0.001.

GMPs differentiate into mature neutrophils through a series of precursors, with a decrease in proliferation capacity at each stage accompanied by the acquisition of lineage-specific features^4, 5, 44^. Because Map3k8 deficiency decreases the cell cycle progression of immature neutrophils after LPS challenge, we next investigated the various neutrophil precursors present in the Ly6G^low^CD11b^+^ BM cell population from non-treated and LPS-treated Wt and Map3k8^−/−^ mice after sorting, cytospin centrifugation and Giemsa-Wright staining. Four different cell types defined based on morphological features were detectable within this BM cell population (Fig. 4A). The distribution of different cell types in Wt and Map3k8^−/−^ cells was very similar in non-treated mice. However, in LPS-treated mice, Map3k8 deficiency decreased the percentage of myelocytes and metamyelocytes, the earliest immature granulocyte precursors, at the expense of an increase in the frequency of banded and mature neutrophils present in the Ly6G^low^CD11b^+^ BM cell population (Fig. 4A). Granule proteins are produced at specific stages of immature neutrophil differentiation. The expression of cathepsin G, a primary marker of granules, decreases during the differentiation of neutrophil precursors^4, 45^. Accordingly, Map3k8^−/−^ Ly6G^low^CD11b^+^ cells contained less cathepsin G mRNA than their Wt counterparts (Fig. 4B). Expression of the transcription factor C/EBPβ is upregulated during emergency granulopoiesis, and this factor is required for the efficient proliferation and differentiation of neutrophil precursors^4, 12, 13^. Map3k8 deficiency significantly decreased C/EBPβ mRNA levels in Ly6G^low^CD11b^+^ cells (Fig. 4B). These findings indicated a requirement for Map3k8 in regulating C/EBPβ expression and maintaining the appropriate proportions of myelocytes and metamyelocytes in the Ly6G^low^CD11b^+^ cell population during LPS-induced emergency granulopoiesis.

### Granulopoiesis in G-CSF-treated Wt and Map3k8^−/−^ mice

**Figure 5:**
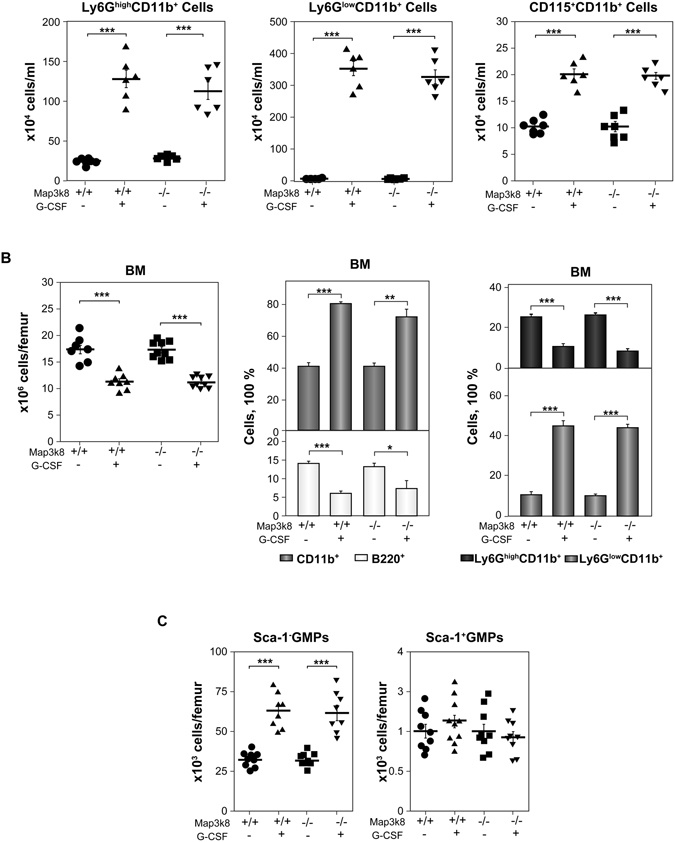
Granulopoiesis in G-CSF-treated Wt and Map3k8^−/−^ mice. (**A**) Wt and Map3k8^−/−^ mice received an i.p. injection of 6 μg of G-CSF (r-metHuG-CSF, Filgrastim) daily on three consecutive days, and circulating myeloid cells were analysed 24 h later. The number of circulating Ly6G^high^CD11b^+^ mature and Ly6G^low^CD11b^+^ immature neutrophils and CD115^+^CD11b^+^ monocytes is shown. The data are presented as the mean ± SEM from two different experiments performed in triplicate. (**B**) Total cellularity of the BM and frequency of BM B220^+^ B and CD11b^+^ myeloid cells and Ly6G^high^CD11b^+^ mature and Ly6G^low^CD11b^+^ immature neutrophils. The frequency of the various cells was calculated relative to total BM cells. (**C**) Isolated BM LIN^−^ cells were subjected to FACS analysis, and the numbers of Sca-1^**−**^ GMPs and Sca-1^**+**^ GMPs per femur were calculated. (**A**–**C)** The data are presented as the mean ± SEM of two or three different experiments performed in triplicate. One-way ANOVA with Newman-Keuls correction was used. *p < 0.05, **p < 0.01, ***p < 0.001.

The increase in G-CSF production in response to LPS challenge is essential for stimulating granulopoiesis^6, 8, 9, 11^. Thus, we next examined the role of Map3k8 in G-CSF-induced myelopoiesis. Wt and Map3k8^−/−^ mice received i.p. injections of recombinant G-CSF on three consecutive days, and circulating myeloid cells were analysed 24 h later. G-CSF increased the number of circulating Ly6G^high^CD11b^+^, Ly6G^low^CD11b^+^, and CD115^+^CD11b^+^ cells to a similar extent in Wt and Map3k8^−/−^ mice (Fig. 5A). In addition, BM cellularity decreased similarly in Wt and Map3k8^−/−^ mice treated with G-CSF; Map3k8 deficiency had no effect on the percentage of BM B220^+^ cells or various analysed myeloid cells (Fig. 5B). G-CSF treatment primarily increased the number of Sca-1^−^ GMPs, but there was no increase in the number of Sca-1^+^ GMPs in BM, and Map3k8 deficiency had no effect on the number of these cells (Fig. 5C). These data indicated that Map3k8 activity is required for the full increase in the number of circulating myeloid cells during LPS-induced emergency granulopoiesis but not for the response to exogenous G-CSF treatment.

### LPS-induced emergency granulopoiesis requires Map3k8 expression in non-haematopoietic cells

**Figure 6:**
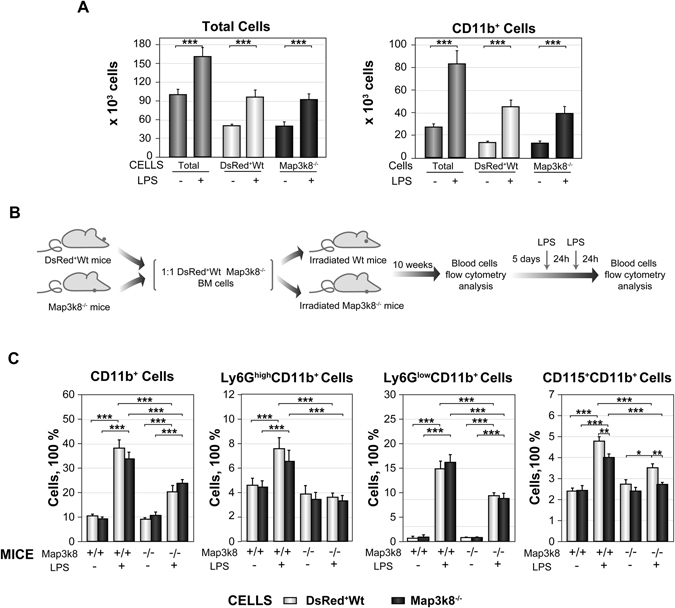
LPS-induced emergency granulopoiesis requires Map3k8 expression in non-haematopoietic cells. (**A**) Isolated BM LIN^−^ cells from DsRed^+^Wt and Map3k8^−/−^ mice were mixed at a 1:1 ratio and used to seed the same MethoCult M3434 plate. Cells were incubated with or without LPS (500 ng/ml) for six days. Total, DsRed+ (Wt) and DsRed^−^ (Map3k8^−/−^) cells were gated and quantified by flow cytometry. The left panel shows the distribution of total cells, and the right panel shows the distribution of CD11b^+^ myeloid cells. Data were obtained from two different experiments performed in quadruplicate, and statistical significance is indicated for differences between DsRed^+^Wt and Map3k8^−/−^ cells with and without LPS treatment only. (**B**) Diagram of the mixed chimaera approach and LPS treatment. (**C**) Circulating DsRed^+^Wt and Map3k8^−/−^ CD11b^+^ myeloid cells, Ly6G^high^CD11b^+^ mature and Ly6G^low^CD11b^+^ immature neutrophils, and CD11b^+^CD115^+^ monocytes from Wt and Map3k8^−/−^ recipient chimaeric mice were analysed before (LPS−) and after (LPS+) LPS treatment. The frequency of the various cells relative to the total number of circulating cells is illustrated (*n* = 8). Statistical significance is shown for differences between the DsRed^+^Wt and Map3k8^−/−^ cell types from Wt and Map3k8^−/−^ recipient mice and between the experimental conditions for Wt and Map3k8^−/−^ recipient mice. (**A**,**C)** DsRed was used to distinguish Wt and Map3k8^−/−^ cells through the use of an extra gate, DsRed *versus* FSC-A, identifying Wt cells as DsRed^+^ and Map3k8^−/−^ cells as DsRed^−^ cells. The data are presented as the mean ± SEM. One-way ANOVA with Newman-Keuls correction was used to compare groups. *p < 0.05, **p < 0.01, ***p < 0.001.

We next investigated the mechanism by which Map3k8 controls LPS-induced granulopoiesis by co-culturing BM LIN^−^ DsRed^+^Wt and Map3k8^−/−^ cells *in vitro* in MethoCult GF M3534 medium. These cells were mixed at a 1:1 ratio and used to seed plates in the presence or absence of LPS. After six days, total cellularity and the number of generated CD11b^+^ cells were analysed by flow cytometry. No differences between DsRed^+^Wt and Map3k8^−/−^ cells were observed under the conditions tested (Fig. 6A). We further investigated whether the decreased LPS-induced granulopoiesis in Map3k8^−/−^ mice was intrinsic to haematopoietic cells by transplanting a 1:1 mixture of BM DsRed^+^Wt and Map3k8^−/−^ cells into irradiated Wt and Map3k8^−/−^ mice to obtain mice with chimaeric BM. After ten weeks, we analysed the frequency of circulating DsRed^+^Wt and Map3k8^−/−^ myeloid cells. After five days, granulopoiesis was induced with LPS, and the circulating myeloid cells were reanalysed (Fig. 6B). Under homeostatic conditions, the percentages of circulating DsRed^+^Wt and Map3k8^−/−^ CD11b^+^, Ly6G^high^CD11b^+^, Ly6G^low^CD11b^+^, and CD115^+^CD11b^+^ cells were similar in both Wt and Map3k8^−/−^ recipient mice (Fig. 6C). LPS treatment significantly increased the frequency of CD11b^+^, Ly6G^high^CD11b^+^, and Ly6G^low^CD11b^+^ cells (both DsRed^+^Wt and Map3k8^−/−^), but this increase was markedly blunted, or completely lost in the case of Ly6G^high^CD11b^+^ cells, when the recipient mice lacked Map3k8. In addition, the percentage of circulating Map3k8^−/−^ CD115^+^CD11b^+^ cells decreased with respect to their Wt counterparts in both Wt and Map3k8^−/−^ recipient mice after LPS treatment and was lower in Map3k8^−/−^ recipient mice (Fig. 6C). Thus, Map3k8 expression in irradiated resistant non-haematopoietic cells, rather than in haematopoietic cells, is important for emergency granulopoiesis, whereas Map3k8 expression in the haematopoietic compartment appears to be required for full induction of the circulating CD115^+^CD11b^+^ monocyte population.

### Map3k8 controls the increase in G-CSF levels in the blood and BM of LPS-treated mice

**Figure 7:**
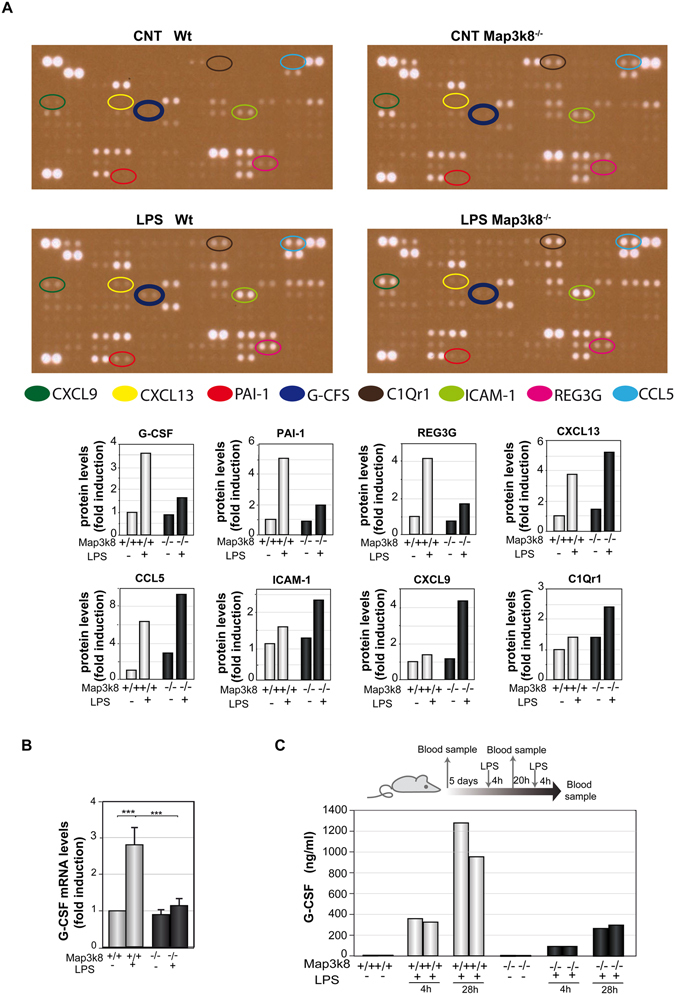
Map3k8 mediates G-CSF production in LPS-induced myelopoiesis. (**A**) Wt and Map3k8^−/−^ mice received injections of LPS or PBS at 0 and 24 h, and BM extracts were obtained 24 h later. Extracts (*n* = 9) from untreated and LPS-treated Wt and Map3k8^−/−^ mice were pooled for analysis of the levels of 111 inflammatory proteins using a mouse XL cytokine array. A 3-min exposure of the arrays is shown. The expression of 8 proteins was modulated by Map3k8 deficiency in LPS-treated mice: chemokine (C-X-C motif) ligand 9 (CXCL9), chemokine (C-X-C motif) ligand 13 (CXCL13), plasminogen activator inhibitor-1 (PAI-1), receptor for complement component C1q (CIQr1), intercellular adhesion molecule 1 (ICAM-1), chemokine (C-C motif) ligand 5 (CCL5), and regenerating islet-derived protein 3 gamma (REG3G). Relative levels of the proteins modulated by Map3k8 are shown. (**B**) RNA was isolated from the BM of mice indicated in (**A**), and G-CSF mRNA levels were assessed by quantitative reverse-transcriptase polymerase chain reaction. The graph shows the mean ± SEM of 4 different experiments, each performed on a pool of cells from 2 animals. One-way ANOVA with Newman-Keuls correction was performed to compare groups. *p < 0.05, **p < 0.01, ***p < 0.001. (**C**) Diagram of LPS treatment of and blood sample collection from Wt and Map3k8^−/−^ mice. The graph shows the circulating G-CSF levels, measured in duplicate from a pool of 3 mice each, at the indicated times after LPS treatment.

Some of the cytokines responsible for regulating steady-state granulopoiesis also increase in abundance during emergency granulopoiesis^5^. In addition, various new signals, primarily other cytokines, also increase under this stress condition and contribute to the massive generation of neutrophils^3, 5, 6, 14–20, 46^. Proteome arrays were used to evaluate the levels of 111 inflammatory proteins in the BM of untreated and LPS-treated Wt and Map3k8^−/−^ mice. Map3k8 deficiency resulted in lower levels of three LPS-induced proteins, namely, G-CSF, plasminogen activator inhibitor-1, and regenerating islet-derived protein 3 gamma. Besides, chemokine (C-X-C motif) ligand 9, chemokine (C-X-C motif) ligand 13, plasminogen activator inhibitor-1, receptor for complement component C1q, intercellular adhesion molecule 1 and chemokine (C-C motif) ligand 5 were more abundant in the BM of LPS-treated Map3k8^−/−^ mice compared with LPS-treated Wt mice (Fig. 7A). Among these proteins, G-CSF plays a major role in steady-state and emergency granulopoiesis^5, 6, 8, 9, 11, 46^. In addition to the lower G-CSF protein levels in BM, G-CSF transcript levels were also strongly decreased in LPS-treated Map3k8^−/−^ mice (Fig. 7B). We also analysed blood G-CSF levels at two different times after LPS treatment (4 and 28 h) and determined that Map3k8 deficiency strongly reduced the circulating levels of G-CSF (Fig. 7C). During emergency granulopoiesis, endothelial cells are the major producers of G-CSF^5, 8^. Accordingly, inhibiting Map3k8 activity in an immortalized murine endothelial cell line markedly blunted G-CSF induction after LPS stimulation. Moreover, the LPS-induced increase in G-CSF mRNA was considerably decreased in a human endothelial cell line (see Supplemental Figure S5).

## Discussion

During life-threating infections, emergency granulopoiesis is triggered to generate an effective response through the massive production of new neutrophils. Both haematopoietic and non-haematopoietic cells that express pattern-recognition receptors sense pathogens and transduce progranulocytic signals to disrupt homeostatic haematopoiesis and trigger massive neutrophil production^6, 47^. We found here that Map3k8 is a determinant factor underlying the increase in G-CSF levels and the generation of full neutrophilia during LPS-induced emergency granulopoiesis. This action required the kinase activity of Map3k8, as a kinase-dead mutant was unable to exert this function. The similar patterns of LIN^−^DsRed^+^Wt and Map3k8^−/−^ cell proliferation and differentiation *in vitro* ruled out a contribution of Map3k8 to the LPS-TLR4 intracellular signalling of HSPCs responsible for their survival, proliferation, and differentiation^48^. Furthermore, the decreased neutrophilia in LPS-treated Map3k8-deficient recipient mice after transplantation of BM Wt and Map3k8^−/−^ cells indicated that Map3k8 expression is required in irradiated resistant non-haematopoietic cells for full neutrophilia during LPS-induced emergency granulopoiesis; this process requires the ability of Map3k8 to mediate the production of granulopoietic signals.

In emergency granulopoiesis, G-CSF production by non-haematopoietic cells and endothelial cells in particular^5, 8, 9^ is crucial for full neutrophilia, although there is evidence for pathogen-specific differences in G-CSF production^5, 6, 9, 11^. Our data showed that Map3k8 is required for attaining high G-CSF levels in both the BM and blood during LPS-induced granulopoiesis, thus directly affecting endothelial cells. In addition, Map3k8 did not affect granulopoiesis in response to exogenous G-CSF, confirming the essential contribution of impaired G-CSF production in Map3k8-deficient mice to the lower levels of neutrophilia during LPS-induced emergency granulopoiesis. G-CSF also maintains neutrophil viability^49^, thereby accounting for the stronger Annexin V^+^ labelling of circulating mature and immature neutrophils in LPS-treated Map3k8^−/−^ mice than in their Wt counterparts.

During emergency granulopoiesis, different and often overlapping cytokines trigger the proliferation and differentiation of HSPCs in the haematopoietic lineage. In systemic infections, interferon γ, TNF-α, and IL-6 induce the expansion of progenitor cell populations, including GMPs expressing Sca-1^18, 42, 43^. G-CSF also accelerates GMP cell cycle progression^5, 6^. Based on these data, we cannot exclude the possibility that Map3k8, in addition to G-CSF, controls the expression of other cytokines that were not detected in the protein array and that may also be involved in maintaining large numbers of GMPs during LPS-induced emergency granulopoiesis. However, G-CSF appears to be unique in instructing these cells to undergo differentiation in a neutrophil-committed lineage rather than in a monocyte-committed lineage^50^ and in inducing the proliferation and differentiation of neutrophil precursors^4, 12, 13, 50^.

During the differentiation of neutrophil precursors from GMPs, each stage is associated with a decrease in proliferation capacity and with the acquisition of lineage-specific features, thus culminating in the production of mature neutrophils^4, 5, 44^. G-CSF accelerates these events by upregulating C/EBPβ in immature neutrophils, and high C/EBPβ levels in neutrophil precursors play a key role in the late stages of emergency granulopoiesis^4, 12, 13^. Our data demonstrated that Map3k8 deficiency slows the cell cycle progression of Ly6G^low^CD11b^+^ BM cells from LPS-treated mice and decreases the proportion of the most immature neutrophils (those with the highest proliferation rates) within this cell population. These findings can be explained by the decreased C/EBPβ levels in Ly6G^low^CD11b^+^ cells after impairing G-CSF expression in the BM of LPS-injected Map3k8^−/−^ mice.

We have recently shown that Map3k8 deficiency increases the apoptosis of monocytes from ApoE^−/−^ mice fed a high-fat diet^51^. The data presented here show that Map3k8 expression in monocytes is required to increase their viability during acute inflammation, thus indicating that the role of Map3k8 in maintaining monocyte viability is not restricted to chronic inflammatory pathologies. However, we cannot ignore the possibility that Map3k8 might perform functions in addition to restricting apoptosis to modulate monocytosis during emergency myelopoiesis.

Neutrophils control the immediate host immune response, and impairment of this response may lead to an extremely high susceptibility to infection. Map3k8^−/−^ mice have been reported to be particularly sensitive to various infections^19, 31–33, 35, 36^ that require a large number of neutrophils for an effective response. We found here that Map3k8 is crucial for the production of large numbers of neutrophils during LPS-induced emergency granulopoiesis through the upregulation of G-CSF levels and the enhanced expansion of granulocyte precursors. Map3k8 controls the production of inflammatory, M1, and M2 cytokines in immune cells^22–24, 28, 32–34^ and has been identified as a potentially interesting target for the treatment of inflammatory diseases, including autoimmune and liver diseases, and diverse types of cancer^37–41^. This newly identified role of Map3k8 as a major player in emergency granulopoiesis should be considered in risk/benefit analyses of therapeutic Map3k8 inhibition and in evaluations of Map3k8 as an anti-inflammatory target.

## Materials and Methods

### Animals and generation of mice with chimaeric bone marrow

Wt, Map3k8^−/−^, and Map3k8^KD^ C57BL/6 J mice were maintained in an animal house in accordance with the Institutional Guidelines for the Care and Use of Laboratory Animals in Research, the relevant European Council Directive *(2010/63/EU)* and Spanish law (R.D. 1201/2005) with approval of the Ethics Committee of the Consejo Superior de Investigaciones Científicas. All the mice used were males that were five weeks old at the time of BM transplantation, and the analyses were conducted ten weeks later. Two weeks before irradiation, recipient Wt and Map3k8^−/−^ mice were given sterilized acidic water supplemented with 10 μg/ml polymyxin B sulphate (Sigma-Aldrich, P4932) and 0.1 mg/ml neomycin (Sigma-Aldrich, N112). This water supply was maintained for five weeks, and the animals were then switched to acidic water without antibiotics for the remainder of the study. BM aplasia was induced by irradiating the mice with one dose of 10 Gy. On the same day, 2 × 10^6^ BM cells (Map3k8^−/−^ and DsRed^+^Wt cells at a 1:1 ratio) were injected into the mice. BM DsRed^+^Wt cells were isolated from C57BL/6 mice transgenic for DsRed expressed under control of the β*-*actin promoter^52^. Ten weeks after BM transplantation, blood was collected from the facial vein, and the percentages of circulating DsRed^+^Wt and Map3k8^−/−^ myeloid cells were determined.

### LPS and G-CSF injections

Mice received two i.p. injections of 30 µg of LPS from *Salmonella enterica serotype typhimurium* (L6511 Sigma-Aldrich) at 0 and 24 h, and the analysis was performed 24 h later. Plasma from non-treated or LPS-treated mice was collected, and G-CSF levels were determined using a Luminex Assay Kit (Thermo-Fisher). Some mice received daily i.p. injections of 6 µg of G-CSF (r-metHuG-CSF, Filgrastim; Amgen) for three consecutive days, and their cells were analysed 24 h later.

### Cell staining for flow cytometry, cell sorting, and Wright-Giemsa staining

BM cells were collected in 50 µl of PBS containing 2 mM EDTA and 0.5% BSA (flow cytometry buffer) by centrifugation of cut femurs at 6000 x *g* for 1 min. Blood was collected in EDTA to prevent clotting. When possible, the cells were incubated before staining with Fc block (anti-CD16/CD32, eBioscience). The antibodies used for flow cytometry and sorting were as follows: from eBioscience: anti-B220 (RA3-6B2), anti-CD11b (M1/70), anti-CD34 (RAM34), anti-CD115 (AFS98), anti-CD117 (ACK2), anti-Ly6G (RB6-8C5), anti-Sca-1 (D7), and anti-CD16/32 (93); from Miltenyi: anti-biotin VioBlue (130-094-669), anti-biotin VioGreen (130-097-022), anti-CD115 (AFS98), anti-CD11b (M1/70), anti-Ly6G (1A8), and anti-Sca-1 (D7). Blood samples were treated for 10 min with VersaLyse Lysing Solution (Beckman Coulter) and washed in flow cytometry buffer. The cell number was determined by addition of Perfect-Count microspheres (Cytognos) to the flow cytometry samples. We distinguished live and dead cells by adding 7-aminoactinomycin D (A1310, Life Technologies) or DAPI (D9542, Sigma-Aldrich) 10 min before FACS analysis. Unstained cells were used as a negative control to establish the flow cytometer voltage settings, and single-colour positive controls were used to adjust compensation. The flow cytometry data were acquired with a FACSCanto II and analysed with FACS Diva software (BD). BM Ly6G^high^CD11b^+^ and Ly6G^low^CD11b^+^ neutrophils were isolated by cell sorting in a FACSAria III cell sorter (BD). Sorted immature neutrophils (150,000) were analysed by cytospin centrifugation, followed by Wright-Giemsa staining according to the manufacturer’s instructions (Hemacolor, Merck).

### Apoptosis, cell proliferation, and cell cycle analysis

Cells labelled with the appropriate antibodies were stained with Annexin V (A1319, Life Technologies) and 7-aminoactinomycin D and immediately subjected to flow cytometry analysis. Cell cycle analysis was performed by treating stained cells with IntraPrep Permeabilization Reagent (Beckman Coulter) and then incubating them with 1 µg/ml DAPI for 20 min at room temperature before analysis. Cell proliferation *in vivo* was assessed by i.p. injection of 200 µg of EDU into mice. BM cells were harvested 18 h later for surface staining. The cells were fixed, permeabilized, and subjected to the Click-iT® reaction (Click-IT Plus ESU Alexa Fluor 647, Molecular Probes) according to the manufacturer’s instructions.

### Quantitative reverse-transcriptase polymerase chain reaction analysis

RNA was extracted from sorted BM Ly6G^low^CD11b^+^ BM cells (9 animals) with the ReliaPrep™ RNA Miniprep System (Promega). RNA was extracted from the BM of femurs from individual animals (*n* = 10) and from endothelial cells (*n* = 4) with Tri Reagent Solution (Invitrogen). The iScript cDNA synthesis kit (Bio Rad) was used for the reverse transcription of 1 μg of RNA. PCR was performed in an Mx3005 P thermocycler (Stratagene) with SYBR Green detection (Bio Rad); the primers are listed in Supplementary Table S1.

### Quantification of inflammatory proteins in the BM

BM was collected from one femur per mouse by centrifugation at 6000 × *g* for 1 min in 50 μl of PBS supplemented with a protease inhibitor cocktail (Roche). Pools of BM from nine femurs were used. The mouse XL cytokine array kit (ARY028, R&D Systems) was used for the simultaneous analysis of the relative expression levels for 111 inflammatory proteins, in accordance with the manufacturer’s instructions. Signals were detected by chemiluminescence and quantified in Photoshop CS5.1 based on densitometry of the X-ray film after exposure for 3 min.

### *In vitro* differentiation of LIN^−^ cell progenitors

Haematopoietic progenitors were isolated from the BM of DsRed^+^Wt and Map3k8^−/−^ mice by magnetic-activated cell sorting (MACS) using a LIN^−^ lineage depletion kit (containing antibodies against CD5, CD11b, GR-1, 7-4, and Ter119) and MACS separation MS columns (Miltenyi). Isolated LIN^−^ DsRed^+^Wt and Map3k8^−/−^ cells (2,500) were plated at a 1:1 ratio in a well with methylcellulose medium, which contains insulin, H transferrin, stem cell factor, IL-3, and IL-6 (MethoCult GF M3534, StemCell Technologies) in the presence or absence of LPS (500 ng/ml). After six days of incubation at 37 °C in an atmosphere containing 5% CO_2_, the cells were harvested and subjected to FACS analysis with Perfect-Count microspheres.

### Cell culture and LPS stimulation

The immortalized mouse cardiac endothelial cell line (MMEC) was cultured in DMEM supplemented with 5% FBS (GIBCO) on collagen. Cells were incubated overnight in 0% FBS and stimulated with 300 ng/ml LPS for 4 h with or without preincubation for 60 min with a Map3k8 inhibitor (C1, 10 μM) that was generously provided by Sir Philip Cohen, and G-CSF levels in the cell supernatant were determined. The human endothelial cell line HMEC-1 was cultured in MCDB131 medium (Corning) supplemented with 10% FBS (GIBCO), 10 ng/ml EGF (E4127, Sigma-Aldrich), 1 µg/ml hydrocortisone (H0888, Sigma-Aldrich), 10 mM glutamine (G7513, Sigma-Aldrich), 100 U/ml penicillin (Gibco, Thermo Fisher Scientific) and 100 µg/ml streptomycin (Gibco, Thermo Fisher Scientific). Cells were incubated overnight in 0.1% FBS and stimulated with 300 ng/ml LPS for 6 h with or without preincubation for 60 min with the Map3k8 inhibitor. The cells were then harvested and subjected to quantitative reverse-transcriptase PCR analysis.

### Statistical analyses

The data are presented as the mean ± SEM. Statistical analyses were performed by one-way ANOVA followed by Newman-Keuls tests using GraphPad Prism software (GraphPad Software, La Jolla, CA). Two-tailed Student’s *t*-tests were used for comparisons between two groups, and p values < 0.05 were considered significant. *p < 0.05, **p < 0.01, and ***p < 0.001.

## Electronic supplementary material

Supplementary Material
