# Supplementary material for: Map3k8 controls granulocyte colony-stimulating factor production and neutrophil precursor proliferation in lipopolysaccharide-induced emergency granulopoiesis
Source: Sci Rep. 2017 Jul 10;7:5010. doi: 10.1038/s41598-017-04538-3 (PMC5503936; doi:10.1038/s41598-017-04538-3)
Supplement: Supplementary file 1 — Supplementary Material [file 41598_2017_4538_MOESM1_ESM.pdf]

Map3k8 controls granulocyte colony-stimulating factor production and neutrophil precursor proliferation in lipopolysaccharide-induced emergency granulopoiesis

Ángela Sánchez, Carlos Relaño, Araceli Carrasco, Constanza Contreras-Jurado, Antonio Martín-Duce, Ana Aranda, and Susana Alemany

A

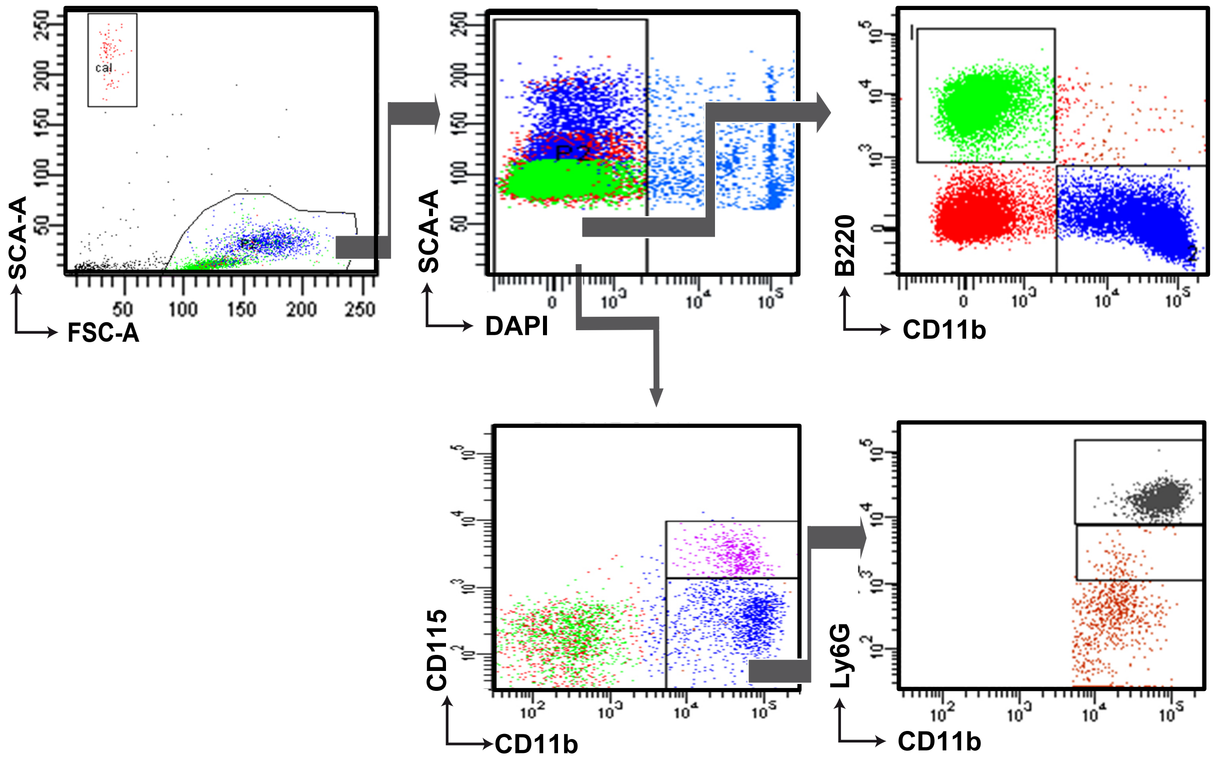
SCA-A
FSC-A
SCA-A
DAPI
B220
CD11b
CD115
CD11b
Ly6G
CD11b

B

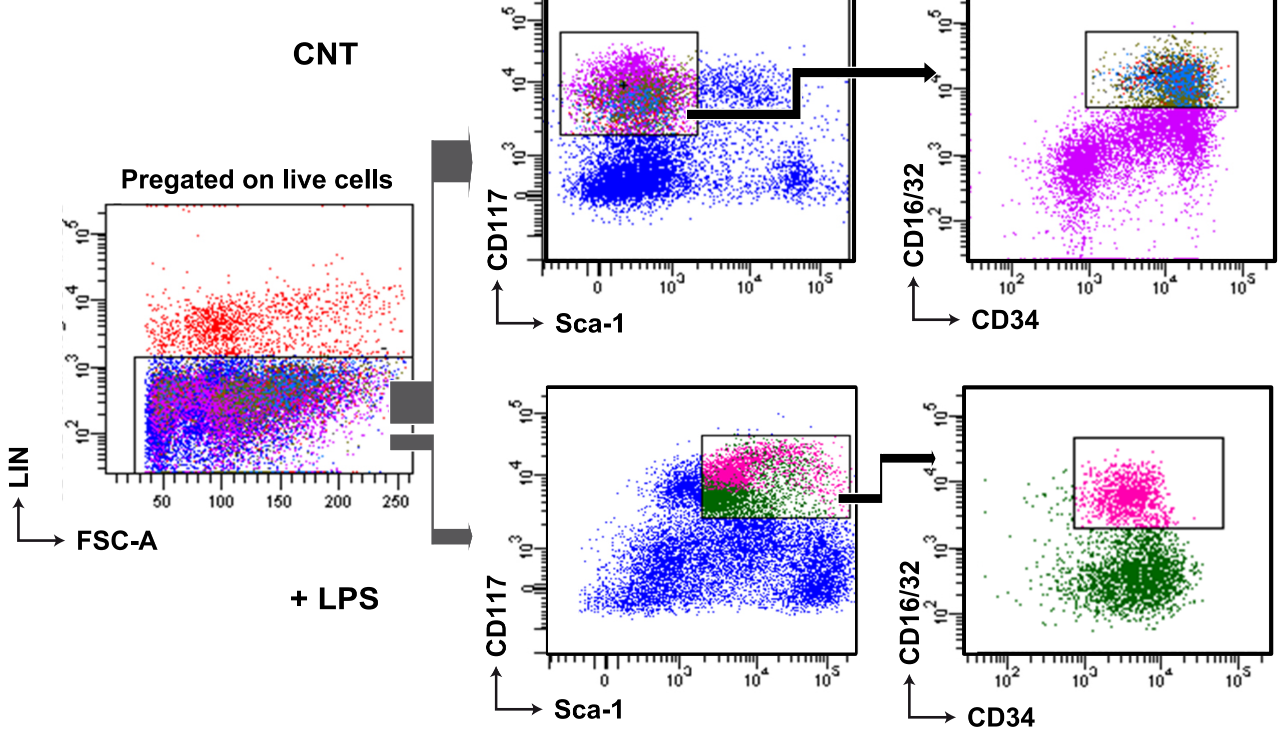
CNT
Pregated on live cells
LIN
FSC-A
+ LPS
CD117
Sca-1
CD16/32
CD34
CD117
Sca-1
CD16/32
CD34

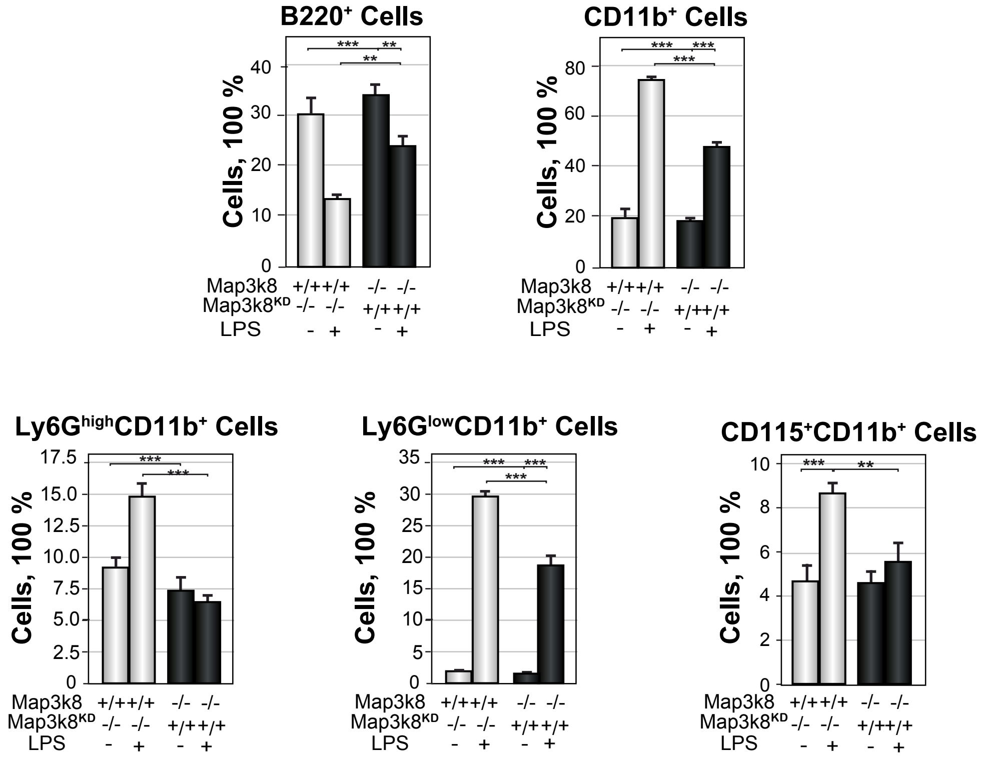

Supplementary Figure 2. Map3k8 activity is required for full LPS-induced emergency myelopoiesis. Wt and Map3k8^{KD} mice received two injections of LPS or PBS and, 24 h after the last injection, circulating B220$^+$ B and CD11b$^+$ myeloid cells, CD11b$^+$Ly6G^{high} mature CD11b$^+$Ly6G^{low} immature neutrophils, and CD11b$^+$CD115$^+$ monocytes were analysed. The frequency of each subset of cells relative to the total circulating cells is shown (mean ± SEM, n=6). One-way ANOVA with Newman-Keuls correction was used to compare groups. Statistical significance is shown as **p<0.01, ***p<0.001.

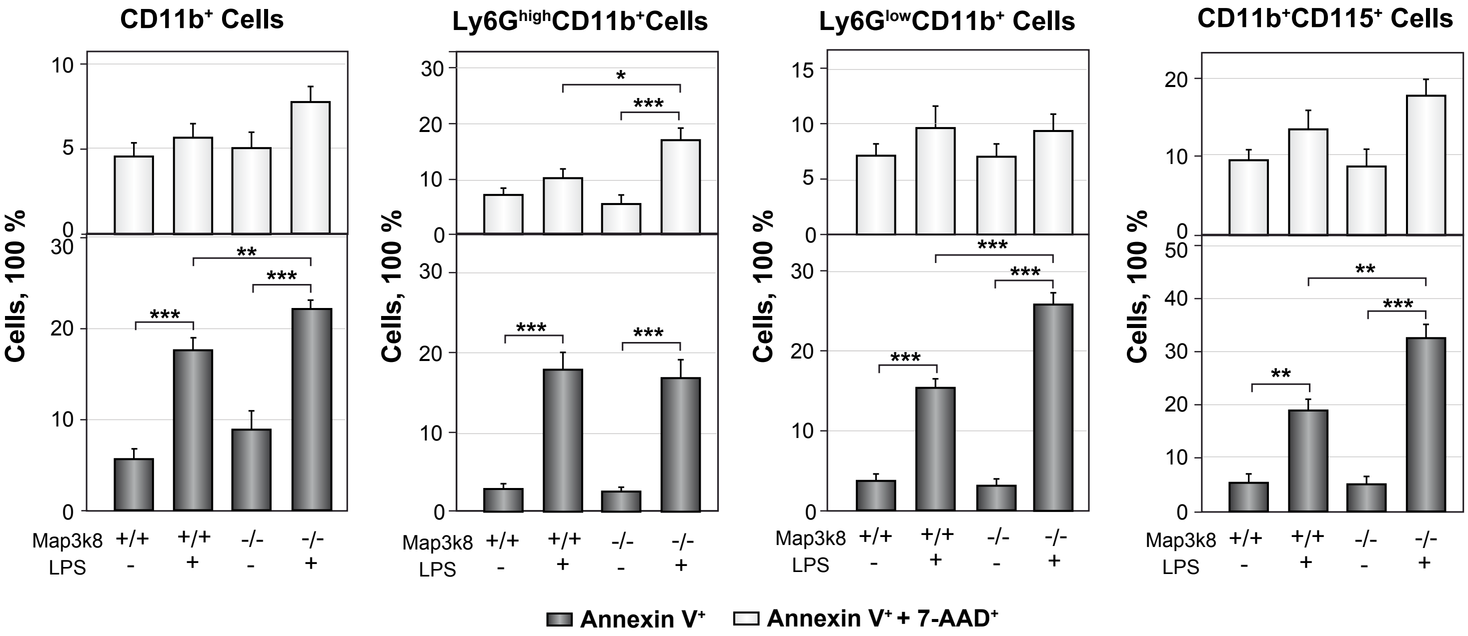

Supplementary Figure 3. Analysis of apoptosis in circulating myeloid cells from LPS-treated Wt and Map3k8$^{-/-}$ mice. Wt and Map3k8$^{-/-}$ mice received two injections of LPS or PBS and, 24 h later, the percentage of Annexin V^{+} and Annexin V^{+} + 7-aminoactinomycin D^{+} cells among CD11b^{+} myeloid cells, Ly6G^{high}CD11b^{+} mature CD11b^{+}Ly6G^{low} immature neutrophils, and CD11b^{+}CD115^{+} monocytes was determined. Graphs show means ± SEM (*n*=6). One-way ANOVA with Newman-Keuls correction was used to compare groups. *p<0.05, **p<0.01, ***p<0.001.

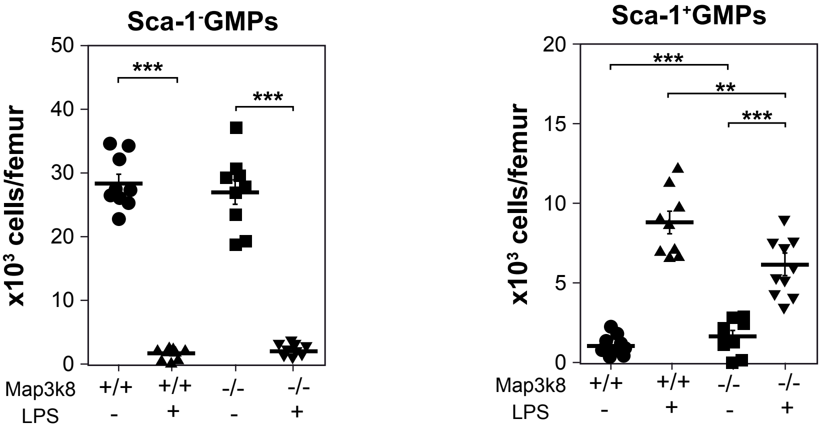

Supplementary Figure 4. Map3k8 deficiency decreases the number of Sca-1$^+$GMPs in LPS-treated mice. Wt and Map3k8$^{-/-}$ mice received injections of LPS and PBS at 0 and 24 h. BM LIN$^-$ cells were isolated 24 h after the last injection and subjected to FACS analysis, and the numbers of Sca-1$^-$GMPs and Sca-1$^+$GMPs per femur were determined. The data shown are means ± SEM (*n*=9). One-way ANOVA with Newman-Keuls correction was used to compare groups. **p*<0.05, ***p*<0.01, ****p*<0.001.

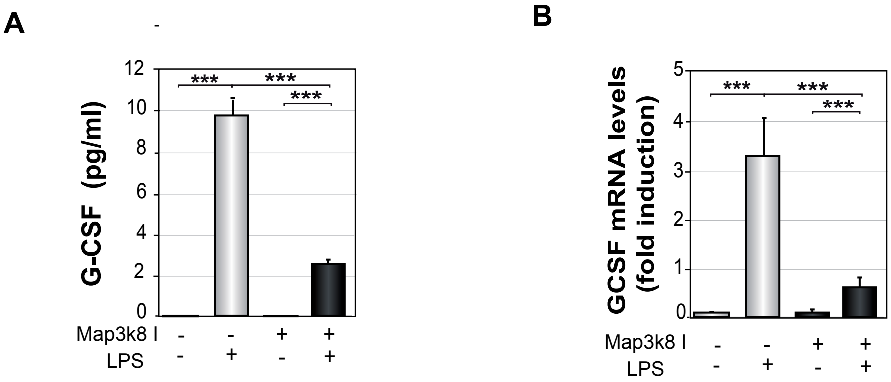

Supplementary Figure 5. LPS-induction of G-CSF is mediated by Map3k8 in endothelial cells. A) G-CSF levels in immortalized murine endothelial cells treated for 4 h with LPS (300 ng/ml) and/or the Map3k8 inhibitor, C1 (5 h, 10 μM). Means ± SEM of 4 different samples each derived from triplicate cultures are shown. **B)** G-CSF transcripts were determined by quantitative reverse-transcriptase polymerase chain in human endothelial cells (HMEC-1) treated with LPS and/or the Map3k8 inhibitor for 6 h. Means ± SEM are shown (n=4). **A,B)** One-way ANOVA with Newman-Keuls correction was used to compare groups. **p<0.01, ***p<0.001.

SUPPLEMENTARY TABLE S1

**Genes**		**Forward Primer (5'-3' sense)**	**Reverse Primer (5'-3' sense)**
Catepsin G	mouse	CAACGGTTCTGGAAAGATGC	CTTCTCGGCCTCCAATGAT
C-EBPβ	mouse	ATCGACTTCAGCCCCTACCT	TAGTCGTCGGCGAAGAGG
G-CSF	mouse	ATGGCTCAACTTTCTGCCCAG	CTGACAGTGACCAGGGGAAC
18-S	mouse	CCAGTAAGTGCGGGTCATAAGC	CCTCACTAAACCATCCAATCGG
G-CSF	human	GAGTGTGCCACCTACAAGCTGTGCC	GGAGAAGCTGGTGAGTGAGTGT
18-S	human	GGGACTTAATCAACGCAAGC	GCAATTATTCCCCATGAACG
